# Noradrenaline and serotonin-dependent sensitization of MSCs to noradrenaline

**DOI:** 10.1016/j.mex.2024.102587

**Published:** 2024-01-28

**Authors:** Vadim I. Chechekhin, Konstantin Yu. Kulebyakin, Natalia I. Kalinina, Pyotr A. Tyurin-Kuzmin

**Affiliations:** Department of Biochemistry and Regenerative Biomedicine, Faculty of Medicine, Lomonosov Moscow State University, Moscow, 119991, Russia

**Keywords:** Calcium imaging at the single cell level, Noradrenaline, Serotonin, Mesenchymal stem cells, Adipose tissue, Obesity, Obesity-associated hypertension

## Abstract

Stem and progenitor cells are characterized by peculiar mechanisms of hormonal regulation. Here we describe a protocol of analysis of hormonal cross-talk in adipose tissue derived multipotent mesenchymal stem cells (MSCs). Specifically, cells were treated by a “sensitizing” hormone/neuromediator followed by the measurement of cellular Ca2+ response to the “readout” hormone after various time intervals. This protocol was successfully used in studies demonstrating a permissive effect of noradrenaline and 5-HT on MSCs sensitivity to noradrenaline, which is a predictive marker of the development of obesity-associated arterial hypertension.

## Graphical abstract

**Figure:**
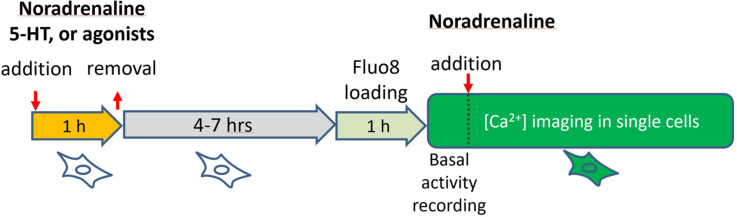

Specifications table

**Table:** 

Subject area:	Biochemistry, Genetics and Molecular Biology
More specific subject area:	Mesenchymal stem cells, obesity-associated arterial hypertension, hormonal regulation
Name of your protocol:	Analysis of hormonal cross-talk in adipose tissue derived multipotent mesenchymal stem cells
Reagents/tools:	Fluo-8-AM green fluorescent calcium binding dye (Abcam, ab142773)
	Fura-2-AM Calcium Indicator cell permeant (ThermoFisher Scientific, F1221)
	Noradrenaline (Abcam, ab120717)
	5-HT (Abcam, ab120528)
	NIS-Elements 5.21.02 (Nikon BioImaging Lab,
	https://www.microscope.healthcare.nikon.com/) or other microscope-based software.
	ImageJ 1.52i (Schneider et al., 2012, https://imagej.nih.gov/ij/)
	Hanks Balanced Salt Solution (HBSS) without phenol red (PanEco, P020)
	HEPES 1 M Solution (HyClone, Cytiva, SH30237.01)
	AdvanceSTEM Mesenchymal Stem Cell Media (HyClone, Cytiva, SH30879.02)
	AdvanceSTEM Supplement (HyClone, Cytiva, SH30878.01), Antibiotic–antimycotic solution (HyClone, Cytiva,
	SV30079.01)
Experimental design:	For the registration of calcium imaging cells were grown in 48-well plate at low density to prevent cell-to-cell communications. To analyze the amount of functionally active cells after pretreatment with noradrenaline or 5-HT we stimulated cells with hormones for 1 h and incubated the cells in full growth medium for an additional 5 h. Cells were loaded with Fluo-8 for 1 h before the registration of calcium responses. To measure the percent of responding cells we recorded the baseline for 5 min then once added noradrenaline. Ca2+ transients were measured in individual cells using an inverted fluorescent microscope. Patients whose MSCs were able to show permissive action of monoamines to noradrenaline were identified by the fact that their MSCs increased their sensitivity to noradrenaline after preincubation with noradrenaline or 5-HT by 1.5 times or more.
Trial registration:	Not applicable
Ethics:	MSCs were isolated from subcutaneous fat tissue of analyzed donors using enzymatic digestion as previously described [3,7]. The work was carried out in accordance with The Code of Ethics of the World Medical Association (Declaration of Helsinki). All donors gave their informed consent and the local ethics committee of the Medical Research and Education Center of Lomonosov Moscow State University (IRB00010587, Moscow, Russia) approved the study protocol (#4, 4 June 2018). The used cell cultures were obtained within the frame of Lomonosov MSU Project "Noah's Ark".
Value of the Protocol:	This protocol was used to discover novel hormonal cross-talks (circuits) in adipose tissue-derived MSCs [4], [5], [6].
	Increase of MSCs sensitivity to noradrenaline by other cAMP-mobilizing hormones is a prognostic marker for the development of obesity-driven arterial hypertension [1,2].

## Description of protocol

Protocol for analyzing the ability of MSCs to sensitize in response to noradrenaline or 5-HT.

1. Prepare in advance stock solutions of 1000x dye (4 mM for both dyes, Fluo-8 or Fura-2), sensitive to the level of intracellular calcium. Aliquots can be stored at −20 °C. Avoid freeze-thaw cycles.
2. Cells 1 day before the experiment are seeded on a culture plate (24-well or 48-well) in low density (the final cell density should be about 10–20 % of a confluent monolayer) (Fig. 1).

**Fig. 1:**
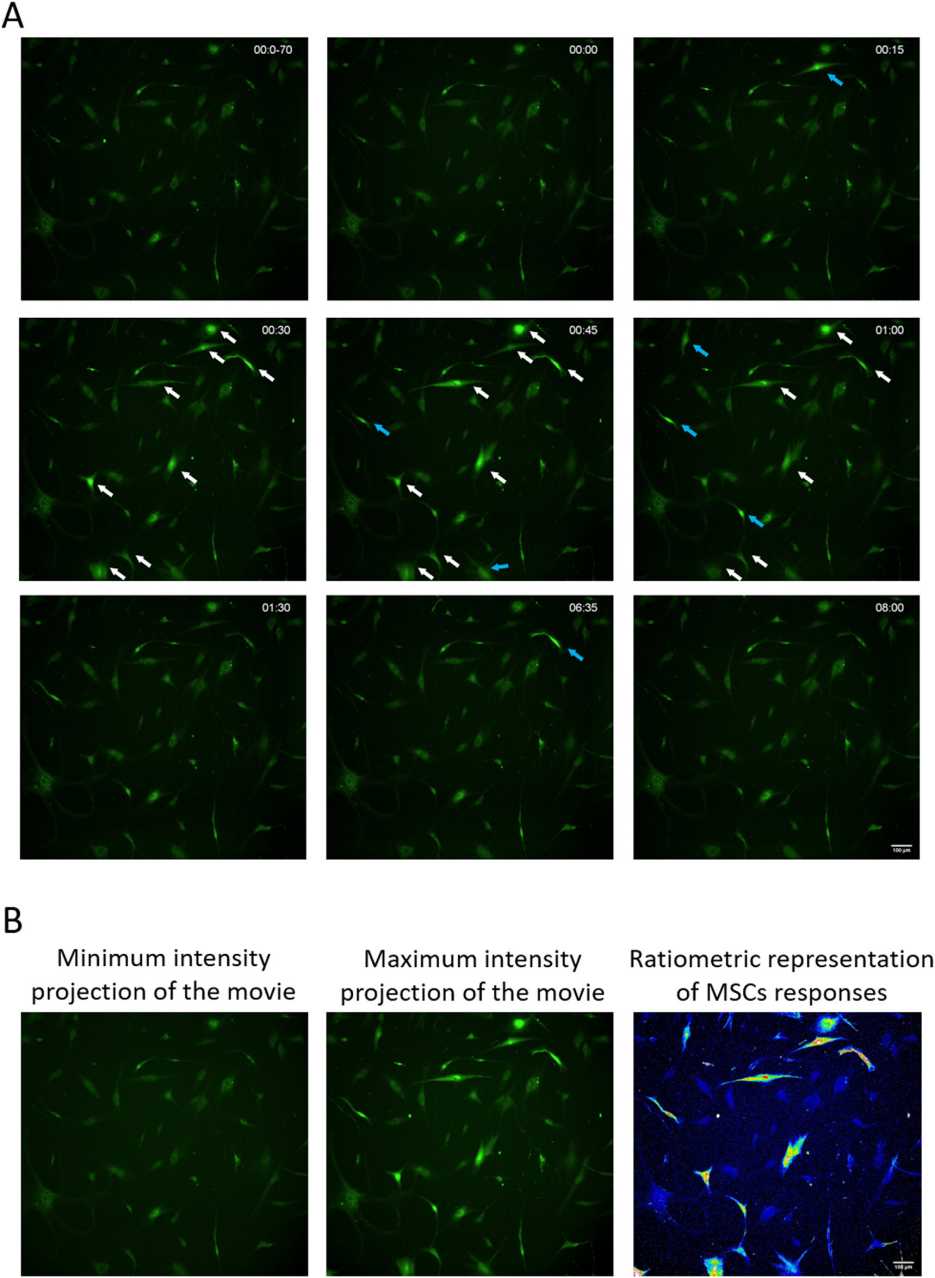
Representative images of MSCs stimulated with noradrenaline (10 µM). (A) – Fluorescent images of MSCs stained with Fluo-8 in different time points before (00:0–70 s and 00:00 time points) and after noradrenaline treatment. Time after noradrenaline addition marked in upper right corner of every image. All arrows point to responding cells; blue arrows point to cells responded not simultaneously with others. Scale bar 100 µm. (B) – An example of presentation of results of measuring calcium levels in cells using ratiometric view.
  *** Cells are grown at low density to prevent cell-to-cell communications during the calcium imaging. The cells should be seeded as evenly as possible. Rotational movements of the plate should be avoided when seeding cells to avoid accumulation of cells in the center of the well.
3. Stimulate the cells with noradrenaline, or 5-HT, or vehicle for 1 h. As vehicle use water in the same volume as noradrenaline, or 5-HT. To do this, prepare 10x solutions of hormones or vehicle (10 µM for noradrenaline and 100 µM for 5-HT) in culture medium and add them directly to the culture medium to the cells. Mix gently, slowly rotating the plate without contact with the surface (to avoid mechanical activation of cell signaling).
4. Wash cells three times with Hanks Balanced Salt Solution (HBSS). All changes of the medium should be performed very carefully to avoid activation of mechanosensitive receptors. Activation of Ca2+ signaling by mechanoreceptors may strongly interfere with hormonal responses.
  *** At all stages of changing the medium before registration of calcium signaling in MSCs, it is extremely important to carry out all manipulations very carefully. MSCs carry mechanosensitive receptors, as a result, calcium signaling is activated in them even due to strong fluid movement. In our experience, after activation of this type of calcium signaling, cells become insensitive to hormones for several hours. As a result, you will not be able to detect a calcium response to the hormone in the experiment. Therefore, all fluid replacements must be done as follows: the old medium is aspirated in such a way that a layer of liquid of 2–3 mm remains above the cells. Smoothly, slowly add a solution to which the medium is replaced. Next, this solution is aspirated the same way, retaining the liquid above the cells, and a new portion is added. This procedure is repeated several times. In the case when it is necessary to wash off the hormone after stimulation of the cells, it must be washed off more thoroughly than the medium with serum (a greater number of repetitions of changing the medium - up to 6–7 times). The cells can be washed multiple times with HBSS and finally twice with complete cell growth medium
5. Change HBSS to full growth medium and incubate the cells for an additional 5 h.
6. Before the experiment, stain the cells for 1 hour using calcium dye – Fura-2 or Fluo-8 (steps 6–8). Cell staining solutions are prepared by diluting 1000x stock solutions in HBSS stabilized with 50 mM HEPES pH 7.2. Working concentrations of dyes are 4 µM for Fura-2, 4 µM for Fluo-8.
  ***We recorded the effect of hormone exposure on the sensitivity of MSCs to noradrenaline using fluorescent dyes that are sensitive to the level of intracellular calcium. For human MSCs, Fura-2 and Fluo-8 have the optimal sensitivity range. Their affinity for calcium (Kd for Fura-2 – 225 nM, for Fluo-8 – 390 nM) is best suited for recording calcium events in human MSCs. Fura-2 and Fluo-8 differ in the wavelength of fluorescence exciting light, allowing them to be combined with a variety of other dyes and fluorescent tags. Fura-2 has two fluorescence excitation peaks with maxima at 340 and 380 nm. The dye fluoresces continuously, and the calcium signal is determined by the ratio of fluorescence intensities at F340/F380. In the case of Fluo-8, the fluorescence of the dye increases in proportion to the level of bound calcium. Fluo-8 fluoresces in a single peak with a fluorescence excitation maximum at 490 nm.
7. Remove the culture medium by washing the cells three times with an excess volume of HBSS stabilized with 50 mM HEPES pH 7.2.
8. Add the prepared dye solution to the cells and place them for 1 hour in an incubator at 37 °C and 5 % CO2 to allow the dye to enter the cells. We do not recommend adding pluronic to staining cells, since, in our experience, it negatively affects the calcium signaling of MSCs.
9. Wash the cells with HBSS stabilized with 50 mM HEPES pH 7.2 to remove any excessive dye.
10. Image cells using an inverted fluorescence microscope. To detect Fluo-8, use a fluorescent light source and a set of filters for green fluorescent protein (GFP) or fluorescein isothyocyanate (FITC). Fura-2 imaging requires a more complex microscope setup: light source capable of rapidly switching fluorescence excitation between 340 nm and 380 nm and corresponding multiband filter sets.
11. During the initial setup of microscope imaging, the priority is to reduce phototoxicity. Accordingly, you should set the camera to the highest sensitivity combined with the lowest possible brightness of the light source. Additionally, it is possible to use binning. Binning refers to the compounding of signal from adjacent pixels in a camera, which increases it's sensitivity, but reduces the resolution of the image.
12. It is optimal to set the time intervals for time-lapse imaging about 20–30 s. We do not recommend taking larger intervals, as calcium peaks may be missed.
13. If microscope setup allows, it is recommended to record in multi-position mode in order to simultaneously register cell responses in experimental wells treated with the hormone and control wells treated with vehicle.
14. After starting the time-lapse recording, the basal activity of MSCs should be recorded for some time (usually, 5–10 min). MSCs from different donors have significantly different basal calcium activity. It is necessary to distinguish spontaneous spikes in intracellular calcium levels from a specific response to noradrenaline.
15. A solution of noradrenaline in HBSS stabilized with 50 mM HEPES pH 7.2 is prepared to be added to the cells at a concentration of 2x (2 µM). Noradrenaline is added directly to the wells of the plate without stopping time-lapse recording. Noradrenaline is added in a volume of medium equal to the volume of medium in which the cells are located. Thus, the final concentration of noradrenaline will be 1x (1 µM). Since the noradrenaline solution is added to the MSCs directly during time-lapse recording, the researcher does not have the opportunity to mix the combined solutions.
16. In cells capable of sensitization in response to preincubation with noradrenaline or 5-HT, the sensitivity of cells to noradrenaline increases [6], and the number of cells responding with calcium to noradrenaline increases [1,2]. The second parameter is more convenient for recording in ordinary experiments. The number of responding cells is calculated as the number of individual cells that formed a calcium response to noradrenaline stimulation, normalized by the total number of cells in the microscope field of view. Due to the variability in the responses of primary cell cultures, it is necessary to perform at least 5–8 independent repeat measurements (different wells of the plate) for each treatment option.

## Protocol validation

**Fig. 2:**
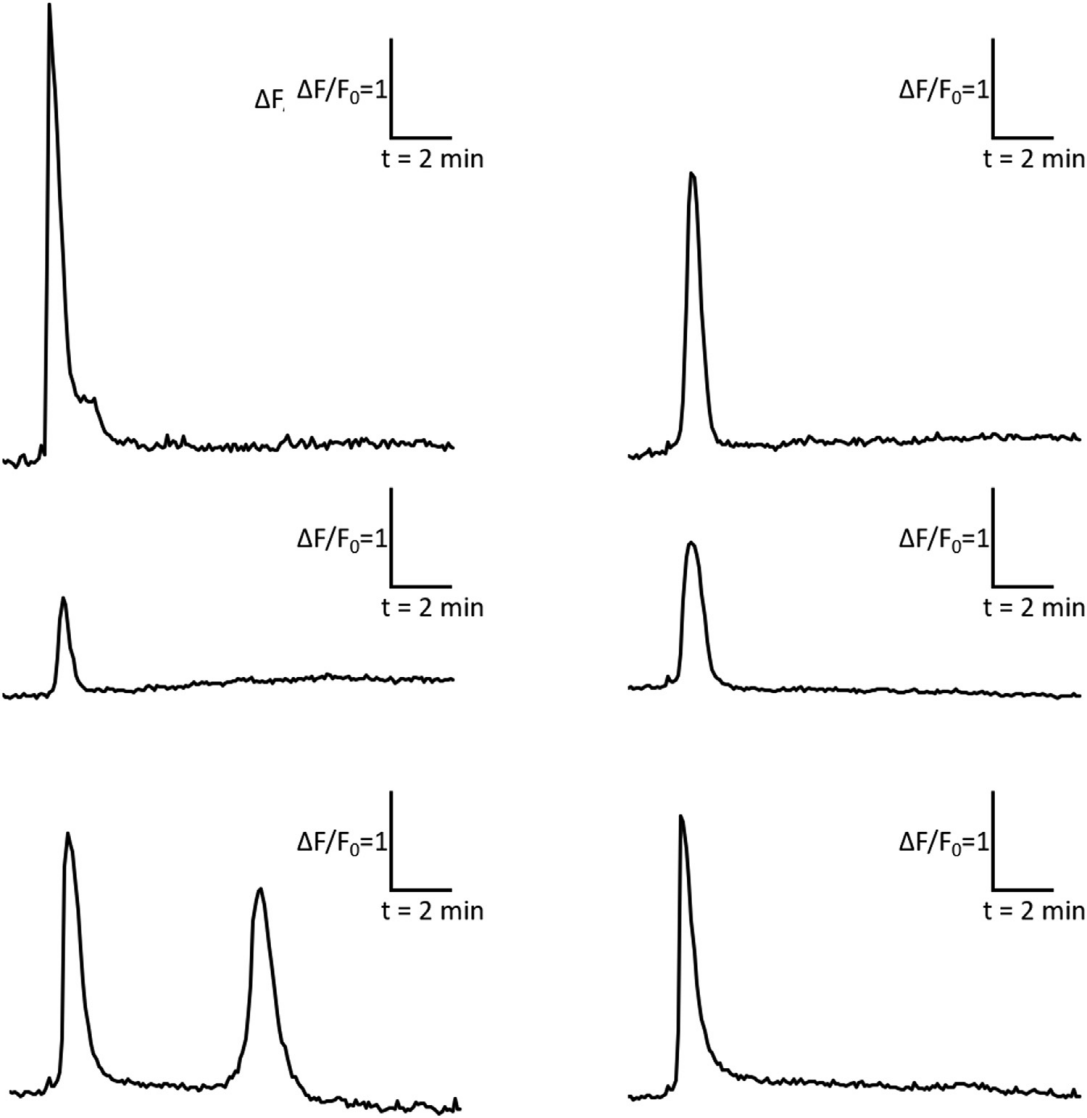
Representative graphs of calcium responses in individual cells measured using Fluo-8 dye. All graphs presented in uniform intensity and time scales. X-axis represents time, while Y-axis represents fold-change in the fluorescence ratio of the individual cell. Each graph shows the change in calcium levels in a single cell.

Typical results of microscope imaging of responding cells are shown in Fig. 1, Fig. 2.

We developed a reliable protocol that allows us to analyze hormonal cross-talks in adipose tissue-derived MSCs. We have tested this protocol in primary cultures of MSCs from adipose tissue from many donors, including those with validated arterial hypertension (*n* = 48). We showed that both noradrenaline and 5-HT, but not histamine, adenosine or dopamine increases the sensitivity of MSCs to noradrenaline [2]. Using this protocol, we showed that noradrenaline specifically acts through β3-adrenoceptors [1], whereas the 5-HT effect is mediated by HTR6 [2]. Stimulation of these receptors in MSCs up-regulates the expression of ɑ1a-adrenoceptors [5] and increases MSCs sensitivity to noradrenaline up to 5 times [6]. Activation of beta3-adrenoceptor or HTR6 has not affected the expression or sensitivity of other Ca2+-mobilizing receptors on MSCs [6].

Clinical data analysis indicated that the extent of this effect correlates with systolic and mean blood pressure of MSCs donors. We compared cells isolated from subcutaneous adipose tissue of hypertensive and normotensive patients with obesity. Noradrenaline and 5-HT increased the portion of cells responding to noradrenaline by activation Ca2+ influx in MSCs isolated from obese hypertensive donors. In contrast, neither hormone was not able to elevate sensitivity to noradrenaline in MSCs isolated from obese normotensive donors. The level of observed increase in the noradrenaline responsiveness was well correlated with mean blood pressure and systolic blood pressure of obese patients, but not diastolic blood pressure [1,2].

## Related research article

Chechekhin et al., Peripheral 5-HT/HTR6 axis is responsible for obesity-associated hypertension, BBA - Molecular Cell Research, 2024, Volume 1871, Issue 2, 119651.

## Funding

This work was supported by 10.13039/501100006769Russian Science Foundation grant #19–75–30007 (https://rscf.ru/project/19–75–30007/).

## CRediT authorship contribution statement

**Vadim I. Chechekhin:** Methodology, Software, Validation, Formal analysis, Data curation. **Konstantin Yu. Kulebyakin:** Validation, Resources, Writing – original draft. **Natalia I. Kalinina:** Conceptualization, Methodology, Resources, Writing – original draft, Visualization, Funding acquisition. **Pyotr A. Tyurin-Kuzmin:** Conceptualization, Methodology, Validation, Formal analysis, Resources, Data curation, Writing – original draft, Visualization, Supervision, Project administration, Funding acquisition.

## Declaration of competing interest

The authors declare that they have no known competing financial interests or personal relationships that could have appeared to influence the work reported in this paper.

## Data Availability

No data was used for the research described in the article.
